# RhoA balances microglial reactivity and survival during neuroinflammation

**DOI:** 10.1038/s41419-023-06217-w

**Published:** 2023-10-20

**Authors:** Renato Socodato, Artur Rodrigues-Santos, Joana Tedim-Moreira, Tiago O. Almeida, Teresa Canedo, Camila C. Portugal, João B. Relvas

**Affiliations:** 1 Institute of Research and Innovation in Health (i3S) and Institute for Molecular and Cell Biology (IBMC), University of Porto, Porto, Portugal; 2 Faculty of Medicine of the University of Porto (FMUP), Porto, Portugal; 3 ICBAS - School of Medicine and Biomedical Sciences, Porto, Portugal

**Keywords:** Microglia, Cell death in the nervous system

## Abstract

Microglia are the largest myeloid cell population in the brain. During injury, disease, or inflammation, microglia adopt different functional states primarily involved in restoring brain homeostasis. However, sustained or exacerbated microglia inflammatory reactivity can lead to brain damage. Dynamic cytoskeleton reorganization correlates with alterations of microglial reactivity driven by external cues, and proteins controlling cytoskeletal reorganization, such as the Rho GTPase RhoA, are well positioned to refine or adjust the functional state of the microglia during injury, disease, or inflammation. Here, we use multi-biosensor-based live-cell imaging approaches and tissue-specific conditional gene ablation in mice to understand the role of RhoA in microglial response to inflammation. We found that a decrease in RhoA activity is an absolute requirement for microglial metabolic reprogramming and reactivity to inflammation. However, without RhoA, inflammation disrupts Ca^2+^ and pH homeostasis, dampening mitochondrial function, worsening microglial necrosis, and triggering microglial apoptosis. Our results suggest that a minimum level of RhoA activity is obligatory to concatenate microglia inflammatory reactivity and survival during neuroinflammation.

## Introduction

Microglia are best known for their immune and protective roles in the CNS. These functions require the orchestrated response of the microglial sensome, from which pattern-recognition receptors (PRRs) on the cell surface recognize pathogen-associated molecular patterns (PAMPs) and tissue damage-associated molecular patterns (DAMPs) as the most critical players. PAMPs, such as NOD-like receptors (NLRs) and Toll-like receptors (TLRs), typically recognize structurally conserved molecules present in various pathogens (e.g., lipopolysaccharides recognition by TLR4) [1]. DAMPs (e.g., ATP, glutamate, DNA, RNA) are released by injured cells, classically related to attracting microglia to damaged sites [1, 2].

Diverse stimuli from pathogens or tissue damage can alter the microglial reactivity state. This alteration is associated with notorious changes in microglial function, with cells transitioning from a ramified to a more amoeboid shape [2–4] and synthesizing and secreting an arsenal of cytokines, chemokines, and other molecular mediators [5]. Transient microglia inflammatory reactivity can help reestablish tissue homeostasis by promoting pathogen elimination, injury repair, and phagocytosis of remaining dead cells [6]. However, failure of compensatory mechanisms [7], loss of “resting signals” [8, 9], or long-term exposure to pathogens or injury [10] may exacerbate microglial immune function, ultimately leading to neuroinflammation and neuronal damage.

Microglia can transit from an acute to a persistent inflammatory state, observed in various neurodegenerative disorders (including Alzheimer’s [11], Parkinson’s [12], and Huntington’s disease [13]. Persistent neuroinflammation is characterized by the exacerbated production of proinflammatory cytokines, chemokines, ROS, and glutamate by CNS glial cells [5]. The accumulation of these mediators in the brain milieu ultimately results in neuronal damage caused by glutamate excitotoxicity [14] and ROS-mediated oxidative stress (via NADPH oxidase) [15]. Moreover, in the most severe cases, proinflammatory cytokines such as TNF, IL-1ß, and IL-6, as well as the chemokines MCP-1 and CCL5 (RANTES), can lead to BBB disruption and peripheral immune cells infiltration [16], worsening both the neuroinflammatory state and tissue damage. Altogether, these events result in a self-perpetuating neuroinflammatory cycle, where neuronal damage induces microglia-mediated production of neurotoxic factors that can, in turn, aggravate neuronal damage, resulting in progressive neurodegeneration.

The extensive morphological changes in microglia transitioning to a more amoeboid cell shape are associated with the remodeling of many intracellular signaling pathways involved in the shift into a proinflammatory phenotype [2, 4]. Consequently, molecular switches coordinating cytoskeletal dynamics and upstream immune-related signal transduction pathways might play an essential role in the proinflammatory polarization of microglia.

Some of those molecules include members of the Rho family that belong to the Ras superfamily of GTP binding proteins ([17], vide [18]). Rho GTPases comprise 20 family members in humans classified into eight subfamilies, including the classical members RhoA, Rac1, and Cdc42 [19]. These molecular switches associate cell-surface receptors (integrins, cadherins, Tyr kinase, cytokine, and G proteins coupled receptors) with the assembly and organization of the actin cytoskeleton, cell polarity, membrane trafficking, and gene transcription in different cell types [20].

Such modulation of cellular functions in response to exogenous stimuli occurs because of the ability of typical Rho GTPases to cycle between an inactive (GDP-bound) and an active (GTP-bound) form, operating as binary switches, which three types of regulatory proteins tightly regulate on and off modes: guanine nucleotide exchange factors (GEFs), GTPase activating proteins (GAPs), and guanine nucleotide dissociation inhibitors (GDIs).

The typical and widely studied Rho GTPase member RhoA regulates the biology of different glial cell populations, including the Schwan cells [21], oligodendrocytes [22], and astrocytes [23]. In microglia, RhoA controls their immune function in the steady state, and its conditional ablation in healthy adult microglia is sufficient to cause neurodegeneration [24]. Thus, RhoA is well-positioned to modulate the microglial state in neuroinflammatory conditions. Here, we investigate the functions of RhoA in the microglial response to inflammation.

## Results

### Inflammation decreases the activity of RhoA in microglia

**Fig. 1:**
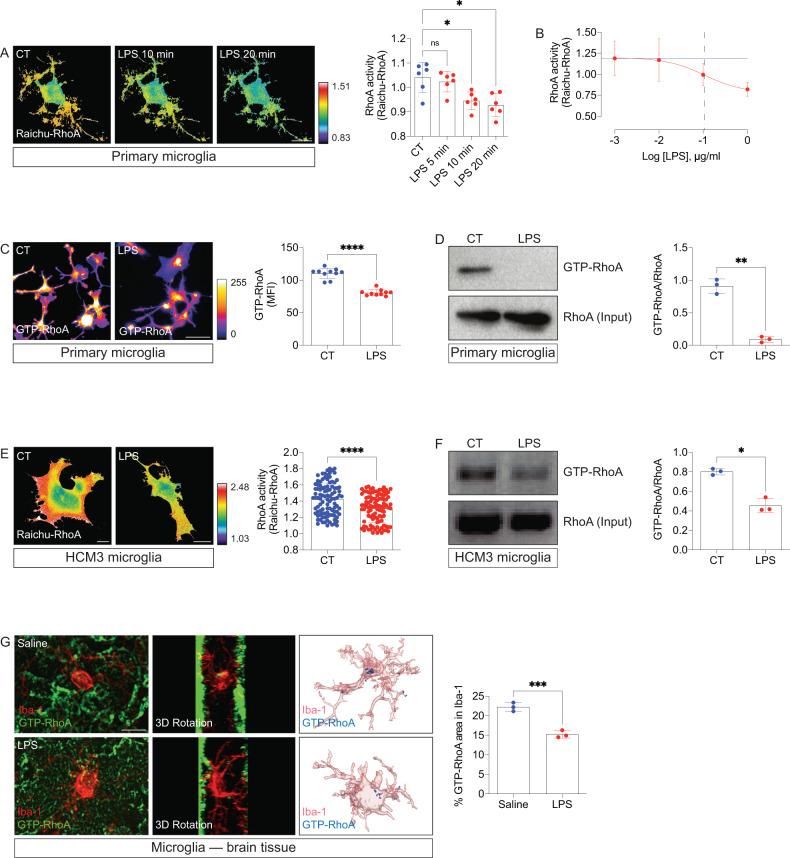
LPS decreases microglial RhoA activity. **A** Primary cortical microglia expressing the Raichu-RhoA biosensor exposed to 1 µg/ml LPS (*n* = 6 cells pooled across 3 independent cultures). Panels represent time-lapse FRET/CFP images coded according to the pseudocolor ramp. Graph (mean and SD) displays FRET/CFP ratio changes normalized at 0 min. *p < 0.05 (One-way ANOVA). **B** Primary cortical microglia expressing the Raichu-RhoA biosensor were exposed to different concentrations of LPS for 20 min (*n* = 10 cells pooled across 5 independent cultures). Graph (mean and SD) displays FRET/CFP ratio changes. The blue line shows untreated cells’ mean FRET/Donor ratio changes. The dashed line indicates the IC_50_. **C**, Primary cortical microglia treated and non-treated (CT) with LPS (1 µg/ml) for 1 h (*n* = 10 independent cultures) and immunostained for GTP-RhoA. Graph (mean and SD) shows amounts of GTP-RhoA as mean fluorescent intensity (MFI). ****p < 0.0001 (Mann-Whitney test). **D** RhoA pull-down on lysates from primary cortical microglia treated and non-treated (CT) with LPS (1 µg/ml) for 1 h (*n* = 3 independent cultures). Graph displays mean with SD. **p < 0.01 (Mann-Whitney test). **E** HMC3 microglia expressing Raichu-RhoA biosensor treated and non-treated (CT) with 1 µg/ml LPS for 1 h (*n* = 100 cells per group from 10 independent experiments). Pseudocolor ramp represents min/max FRET/CFP ratios. Graph (mean and SD) displays FRET/CFP ratio changes. ****p < 0.0001 (unpaired t-test). **F** RhoA pull-down on lysates from HCM3 microglia treated and non-treated (CT) with LPS (1 µg/ml) for 1 h (*n* = 3 independent cultures). Graph displays mean with SD. *p < 0.05 (Mann-Whitney test). **G** Immunofluorescence images of GTP-RhoA and Iba-1 on cortical tissue sections from brain cortex of adult mice injected with saline or LPS (4 mg/Kg; 24 h). Graph (mean and SD) shows amounts of GTP-RhoA in Iba-1+ cells (*n* = 3 mice per group). *p < 0.001 (paired t-test). Scale bars: 10 µm (**A,** **C**, and **G**); 20 µm (**E**).

In many biological systems, cellular and tissue homeostasis require tight regulation of the RhoA activation [25]. We examined whether microglial inflammation modulates the activation status of RhoA. We used lipopolysaccharide (LPS), a classical immune-related stimulus that induces a robust reactivity response in the microglia [26]. We first measured RhoA activity by FRET using the Raichu-RhoA biosensor [27] in primary cortical microglia exposed to LPS. This FRET-based approach allows precise monitoring of the RhoA activation/inactivation cycle depending on the binding of either GDP or GTP. Living primary cortical microglia exhibited a significant reduction of RhoA activity following 10 min of exposure to LPS (Fig. 1A). A dose-response curve revealed an IC_50_ of 110 ng/ml for RhoA inhibition after 20 min exposure to LPS (Fig. 1B). Then we performed immunofluorescence imaging with a GTP-RhoA antibody (detecting active RhoA) on primary cortical microglia after LPS treatment. As expected, we found a significant decrease in GTP-RhoA amounts in primary cortical microglia treated with LPS compared with controls (Fig. 1C). In addition, we carried out pull-down assays to detect the amounts of active (GTP-bound) RhoA in lysates from primary cortical microglia. In agreement with the FRET and immunofluorescence data, LPS substantially decreased GTP-RhoA amounts relative to control lysates (Fig. 1D). We also measured RhoA activity in the HMC3 microglial cell line following LPS exposure. HMC3 microglia treated with LPS consistently showed a significant decrease in RhoA activity (measured by FRET with the Raichu-RhoA sensor (Fig. 1E) or by RhoA pull-downs (Fig. 1F)) compared with control cells. To verify if the activity of RhoA also decreased in microglia in vivo, we used double-labeling immunofluorescence with Iba-1 and the GTP-RhoA antibody (detecting active RhoA) coupled to high-resolution confocal imaging in cortical tissue sections from brains of adult mice following neuroinflammation triggered by a single systemic administration of LPS (4 mg/kg). We found significantly decreased GTP-RhoA in Iba-1^+^ cells in the brains of LPS-treated mice compared with those treated with saline (Fig. 1G).

### RhoA activation limits microglia metabolic reprogramming and inflammatory reactivity

After showing that microglia inflammation induced by LPS was associated with decreased RhoA activity, we evaluated if preventing such reduction by artificially sustaining RhoA activity would impact the microglial response to LPS. We focused on two aspects of the microglial inflammation process: [1] microglia metabolic reprogramming and [2] microglia proinflammatory polarization.

Proinflammatory stimuli, such as LPS, induce microglia metabolic reprogramming by promoting a shift from oxidative phosphorylation to glycolysis [28, 29]. Therefore, since microglia activity status impacts their metabolism, we questioned whether sustained RhoA activity would prevent metabolic reprogramming and inflammation induced by LPS. To answer that, we focused on some of the most significant indicators of cellular metabolic reprogramming: ATP/ADP balance, glucose consumption, and pyruvate and lactate levels.

**Fig. 2:**
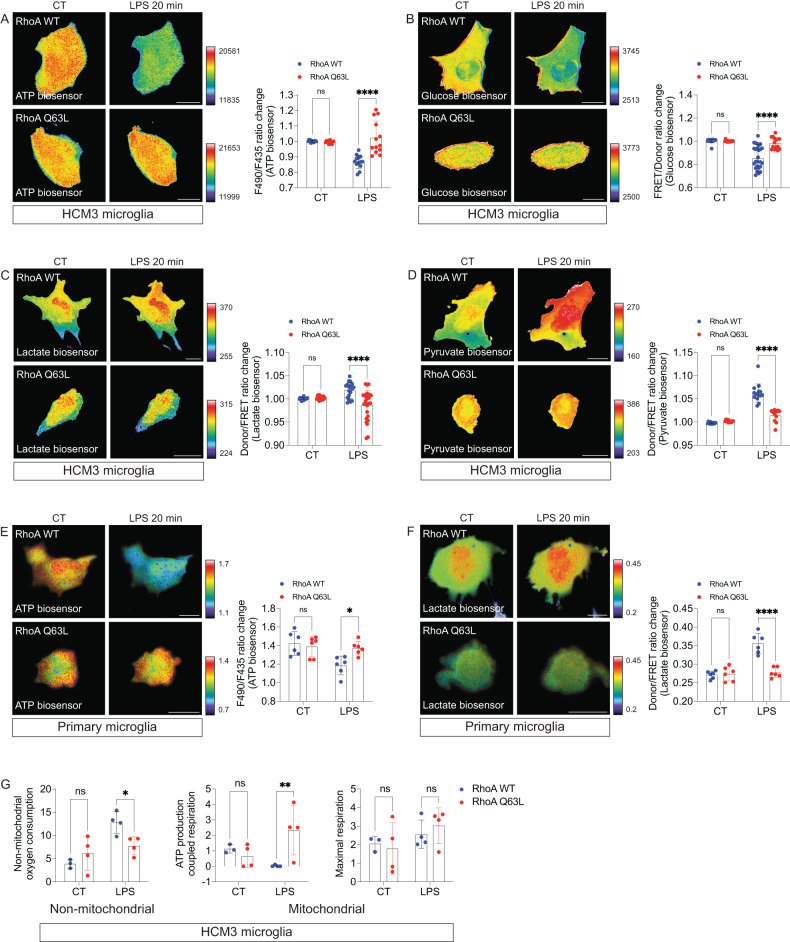
RhoA regulates microglial metabolic reprogramming during inflammation. HMC3 microglia expressing the ATP biosensor (**A**), Glucose biosensor (**B**), Lactate biosensor (**C**), or Pyruvate biosensor (**D**) were transfected with RhoA Q63L (red) or RhoA WT (blue) and exposed to LPS (1 µg/ml; 20 min) (*n* = 15-30 cells per group from 3 independent experiments for each biosensor). Primary cortical microglia expressing the ATP biosensor **(E)** or Lactate biosensor (**F**) were transfected with the RhoA Q63L (red) or RhoA WT (blue) and exposed to LPS (1 µg/ml; 20 min) (*n* = 6 cells per group from 3 independent experiments for each biosensor). Panels are time-lapse ratio images coded according to the pseudocolor ramps. Graphs (means and SD) display F490/F435 (**A** and **E**), FRET/Donor (**B**), and Donor/FRET (**C,** **D**, and **F**) ratio change at 0 (CT) and 20 min. **G** Seahorse measurements of bioenergetic parameters in HCM3 microglia expressing RhoA Q63L or RhoA WT. The parameters were calculated based on the OCR following the sequential addition of LPS, oligomycin, FCCP, rotenone, and antimycin A. Results are from at least 3 independent experiments. Graphs show the mean with SD. *p < 0.05, **p < 0.01, ***p < 0.001, ****p < 0.0001 (Two-way ANOVA). Scale bars: 20 µm.

ATP/ADP balance represents the ratio between ATP generation and expense (by conversion into ADP) and is decreased in metabolic processes that are less energetically efficient, such as glycolysis [30]. We co-transfected HMC3 microglia with a constitutively active RhoA mutant (RhoA Q63L) and an ATP/ADP ratiometric biosensor to monitor variations in ATP levels after challenging microglia with LPS. Live-cell imaging showed that in microglia overexpressing wild-type RhoA (RhoA WT), LPS exposure decreased ATP levels, i.e., increased ATP consumption (Fig. 2A). Such reduction in ATP was prevented by overexpressing the RhoA Q63L mutant in microglia (Fig. 2A).

Glucose fuels most microglial metabolic pathways and is critically involved in mitochondrial ROS production via the electron transport chain. However, glucose consumption increases rapidly during glycolysis to provide sufficient ATP and NADH required for adequate cell functioning [31]. To evaluate glucose consumption (disregarding its uptake), we performed live-cell imaging assays in HMC3 microglia co-expressing the mutant RhoA Q63L and a FRET-based glucose biosensor [32] in a glucose-free medium. In line with what we observed with the ATP/ADP balance, in microglia overexpressing RhoA WT, LPS treatment decreased glucose levels, i.e., increased glucose consumption, an effect prevented in microglia overexpressing the constitutively active mutant RhoA Q63L (Fig. 2B).

Pyruvate and lactate are the most relevant glycolysis by-products. Pyruvate directly results from the glycolytic breakdown of glucose, generating energy in the cell [31]. Afterward, lactate dehydrogenase reduces pyruvate into lactate, accumulating in the cytosol and serving as the glycolytic metabolism’s direct output [33]. To evaluate if sustained RhoA activity impacts either pyruvate or lactate levels after LPS stimulation, we co-transfected HMC3 microglia with RhoA Q63L and either pyruvate or lactate FRET biosensors [34, 35]. Live-cell imaging showed that LPS increased the amounts of both by-products in the cytosol of microglia overexpressing RhoA WT (Fig. 2C and D). However, such a decrease was blocked in cells overexpressing RhoA Q63L (Fig. 2C and D). We confirmed in primary cortical microglia the effect of constitutively active mutant RhoA Q63L in inhibiting the LPS-induced decrease of ATP levels (Fig. 2E) and LPS-induced increase of cytosolic lactate (Fig. 2F).

To further strengthen the energy metabolism data, we conducted a Seahorse assay to measure the bioenergetic profile of intact HCM3 microglia expressing either RhoA WT or RhoA Q63L during inflammation. We found that when in control conditions, HCM3 microglia expressing RhoA WT and RhoA Q63L showed similar levels of non-mitochondrial oxygen consumption, mitochondrial ATP production coupled to respiration, and maximal mitochondrial respiratory capacity (Fig. 2G). When LPS was introduced to RhoA WT microglia, it increased non-mitochondrial oxygen consumption and decreased mitochondrial ATP production related to respiration without affecting the maximal mitochondrial respiration (Fig. 2G). This confirmed that LPS promotes glycolysis to reprogram the energetic metabolism of RhoA WT microglia. However, this LPS-induced glycolytic reprogramming did not occur in RhoA Q63L microglia (Fig. 2G). These results suggest that microglial metabolic reprogramming during inflammation requires a reduction of RhoA activity.

Microglia inflammatory polarization is classically associated with the response to a proinflammatory stimulus, such as LPS [36]. Besides morphological alterations, several physiological changes characterize this response, culminating in the production of many inflammatory mediators that drive the progress of inflammation [5]. Typically, LPS-activated microglia produce high levels of reactive oxygen species (ROS) associated with the activation of intracellular protein kinases, including Src kinase [37], AMP-activated protein kinase (AMPK), and extracellular signal-regulated kinases (ERKs), culminating with the activation of the master proinflammatory transcription factor NF-κB [38, 39]. Thus, we questioned whether the decrease of RhoA activity triggered by LPS would alter those classic hallmarks of microglia proinflammatory activation.

**Fig. 3:**
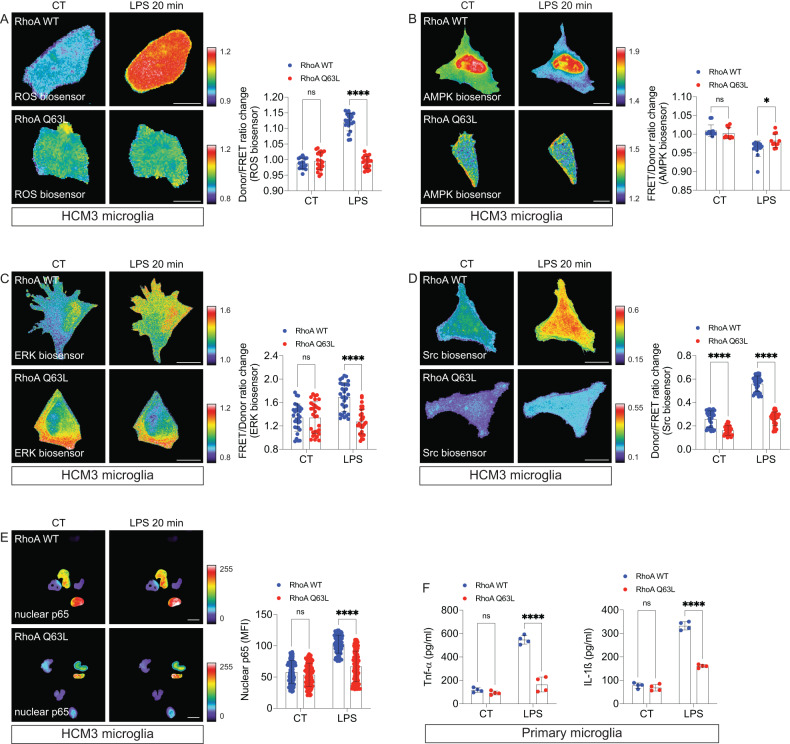
RhoA regulates microglial proinflammatory reactivity. HMC3 microglia expressing a ROS biosensor (**A**), AMPK biosensor (**B**), ERK biosensor (**C**), Src biosensor (**D**), and GFP-tagged p65 NFkB subunit (**E**) were transfected with the RhoA Q63L construct or with RhoA WT and then exposed to LPS (1 µg/ml for 20 min) (*n* = 18–100 cells per group from 3 independent experiments for each biosensor). Panels show time-lapse ratio images or mean fluorescent intensity (MFI) coded according to the pseudocolor ramps. **F** ELISA (TNF-α or IL-1ß) from culture supernatants of primary cortical microglia transfected with RhoA Q63L or RhoA WT and exposed to LPS (1 µg/ml) for 3 h (*n* = 4 independent experiments). Graphs are means with SD. *p < 0.05, ***p < 0.001, ****p < 0.0001 (Two-way ANOVA). Scale bars: 20 µm.

ROS are one of the main factors produced by inflammatory microglia as secondary messengers, which can modulate inflammatory gene expression [40] and as inflammatory molecules [41]. ROS in microglia are mainly produced in mitochondria and the plasma membrane (via NADPH oxidase) [40]. To evaluate if sustained RhoA activity would impact microglial ROS production during LPS stimulation, we used the RhoA mutant Q63L in HMC3 microglia, co-expressing the ROS FRET biosensor HSP. As expected, RhoA WT cells exhibited a robust increase in ROS production following LPS treatment (Fig. 3A). However, ROS production was prevented entirely in microglia expressing the RhoA mutant Q63L (Fig. 3A). The effect of RhoA Q63L in preventing the LPS-induced increase of ROS production was further confirmed using the oxidative stress-related dye dihydroethidium in primary microglia (Suppl. Fig. 1A).

AMPK, more than a master metabolic regulator, is crucial in the microglia’s inflammatory response [42]. Its activation suppresses LPS-induced secretion of proinflammatory mediators [43, 44]. We asked whether RhoA activation would modulate AMPK activity. Thus, we co-transfected HMC3 microglia with RhoA Q63L and, in this case, an AMPK activity FRET biosensor. Results showed that LPS decreased AMPK activation in RhoA WT cells, an effect significantly attenuated in cells expressing the constitutively active mutant RhoA Q63L (Fig. 3B).

In immune cells, signaling by ERK is essential for the production of inflammatory mediators [45, 46]. In microglia, the activity of ERK controls the production and secretion of the inflammatory cytokine IL-1ß [47]. Thus, we investigated the role of RhoA in ERK activation upon exposure to LPS. As expected, LPS triggered ERK activation (accessed using the ERK biosensor EKAR) in living HCM3 microglia overexpressing RhoA WT (Fig. 3C). However, overexpressing the mutant RhoA Q63L prevented the LPS-mediated ERK activation (Fig. 3C). The effect of RhoA Q63L in preventing the LPS-induced increase of ERK activation was further confirmed by Western blotting in HCM3 microglia using an antibody recognizing the active form of ERK (Suppl. Fig. 1B).

The Src family kinases (SFKs) are a family of non-receptor protein tyrosine kinases from which the proto-oncogene Src is the archetype member [48]. The activity of SFKs, including Src, regulates innate immunity [49] and inflammation [50]. When activated, Src controls microglial response via increased production and secretion of proinflammatory cytokines and ROS [37, 51]. Moreover, decreasing RhoA activity is sufficient to increase Src activation in the microglia [24]. Therefore, we performed FRET-based live-cell imaging with an Src biosensor to evaluate if constitutively active RhoA modulates Src activation during LPS exposure. Results showed that in RhoA WT cells, LPS exposure increased Src activity. Still, the overexpression of RhoA Q63L completely blocked this effect (Fig. 3D). The impact of RhoA Q63L in preventing the LPS-induced increase of Src activation was further confirmed by Western blotting in HCM3 microglia using an antibody recognizing the active form of Src (Suppl. Fig. 1C).

Upstream signaling by ROS [52], AMPK [53], ERK [54], and Src [55] converge to modulate NF-κB activation, leading to downstream proinflammatory responses in immune cells. Thus, we measured the nuclear accumulation of the p65 subunit of the NF-κB complex (a readout of NF-κB activation) in microglia overexpressing RhoA. Live-cell imaging showed that while LPS elicited a robust increase of GFP-tagged p65 subunit in the nucleus of HCM3 microglia overexpressing RhoA WT, the overexpression of RhoA Q63L prevented this LPS effect entirely (Fig. 3E). Moreover, overexpression of RhoA Q63L in primary cortical microglia prevented the secretion of the NF-κB-regulated proinflammatory cytokines Tnf-α and IL-1ß elicited by LPS (Fig. 3F).

These data strongly correlate with our previous work in which the decrease of RhoA activity in steady-state microglia elicits Src and NF-κB activation, leading to a proinflammatory polarization of the microglia [24]. In line with these previous results, and in contrast with the data using the RhoA Q63L mutant, primary microglial cultures overexpressing the dominant negative (T19N) RhoA mutant had increased secretion of Tnf-α and IL-1ß compared to cultures overexpressing WT RhoA that was further increased upon LPS exposure (Suppl. Fig. 1D), suggesting that dysregulation of RhoA activity can either attenuate or exacerbate microglial response during inflammation.

The results based on two different aspects of microglia inflammation (Figs. 2 and 3) suggest that a decrease in RhoA activity is an absolute requirement for the microglial inflammatory reactivity induced by LPS.

### Neuroinflammation worsens microglial cell death in the absence of RhoA

Having shown that a decrease in RhoA activity is crucial for microglia to initiate an inflammatory response, we then questioned whether the complete lack of RhoA would poise this response. We used complementary in vivo and in vitro methodologies in microglia exposed to LPS to do that.

**Fig. 4:**
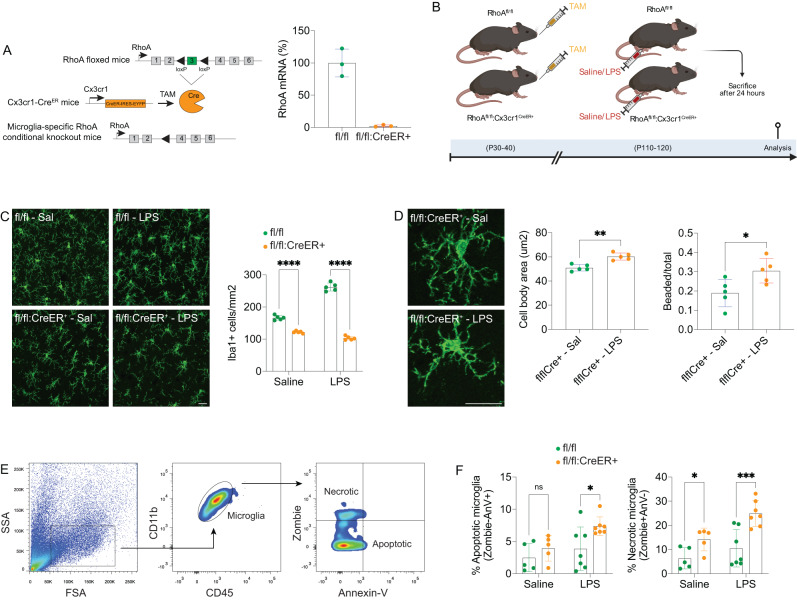
Neuroinflammation causes microglial cell death in the absence of RhoA. **A** qRT-PCR validation of loss of RhoA expression in flow-cytometry sorted microglia from RhoA^fl/fl^ and RhoA^fl/fl^:Cx3cr1^CreER+^ mice after tamoxifen administration (*n* = 3 mice per genotype). **B** Schematics for LPS-induced neuroinflammation. **C** and **D** Immunofluorescence images of Iba-1 on cortical tissue sections from brain cortex of RhoA^fl/fl^ or RhoA^fl/fl^:Cx3cr1^CreER+^ mice after tamoxifen administration and injected with saline or LPS (4 mg/Kg; 24 h). Graphs (mean and SD) show Iba-1+ cell counting and microglial morphological analyses (*n* = 5 mice per group). *p < 0.05, **p < 0.01, ***p < 0.001 (Two-way ANOVA). Scale bars: 20 µm. **E** FACS gating strategy for detecting microglial necrosis and apoptosis. **F** FACS analyses in brain microglia from RhoA^fl/fl^ or RhoA^fl/fl^:Cx3cr1^CreER+^ mice after tamoxifen administration and injected with saline or LPS (4 mg/Kg; 24 h). Graphs (mean and SD) show the percentage of necrotic or apoptotic microglia (*n* = 5–7 mice per group).

For the in vivo approach, we performed conditional ablation of RhoA in adult microglia (as we did before [24]) by crossing RhoA floxed mice (RhoA^fl/fl^; control) with mice expressing both EYFP and tamoxifen-inducible Cre recombinase (Cre^ER^) under the endogenous regulation of Cx3cr1 promoter (Cx3cr1^CreER-IRES-EYFP^) [56, 57]. Confirming previous results [24], following tamoxifen administration, RhoA gene inactivation effectively occurred in microglia from RhoA^fl/fl^:Cx3cr1^CreER+^ mice (Fig. 4A). Overall, this inducible model allows us to adjust the timing of microglial RhoA gene inactivation to our specific experimental requirements.

To evaluate if the lack of RhoA impacts microglial response during neuroinflammation, RhoA^fl/fl^ and RhoA^fl/fl^:Cx3cr1^CreER+^ mice were systemically administered with LPS (4 mg/kg) or saline at P100-P110 and their brains were analyzed 24 h later (Fig. 4B).

We previously demonstrated that lack of RhoA in the steady state causes microglial necrosis, leading to cell loss [24]. Such increased microglial cell loss was confirmed comparing saline-treated RhoA^fl/fl^ and RhoA^fl/fl^:Cx3cr1^CreER+^ brains (Fig. 4C). Moreover, confocal imaging coupled to immunofluorescence with Iba1 (a microglial marker) on brain tissue sections revealed that LPS further decreased Iba1^+^ cells in RhoA^fl/fl^:Cx3cr1^CreER+^ brains compared to saline-treated RhoA^fl/fl^:Cx3cr1^CreER+^ brains (Fig. 4C). Compared to saline-treated RhoA^fl/fl^ brains, LPS-treated RhoA^fl/fl^ brains displayed, as expected, a significant expansion of Iba1^+^ cell population indicative of microgliosis (Fig. 4C). In addition, morphological analyses of the remaining Iba1^+^ microglia in brain sections from LPS-treated RhoA^fl/fl^:Cx3cr1^CreER+^ mice revealed a significant increase of microglial process shortening and beading (a hallmark of cell damage) associated with abnormal cell swelling compared to saline-treated RhoA^fl/fl^:Cx3cr1^CreER+^ littermates (Fig. 4D). To reinforce the confocal data, we performed flow cytometry with cell death markers to assess the percentage of necrotic (Zombie^+^AnV^-^) and apoptotic (Zombie^-^AnV^+^) microglia present in RhoA^fl/fl^ and RhoA^fl/fl^:Cx3cr1^CreER+^ brains (Fig. 4E). Similar to what we previously reported [24], we observed increased necrosis but not apoptosis in microglia lacking RhoA in control conditions. As expected, we found that in RhoA^fl/fl^ microglia, neuroinflammation did not cause necrosis or apoptosis. Still, in microglia RhoA-deficient mice (RhoA^fl/fl^:Cx3cr1^CreER+^), neuroinflammation aggravated cytotoxicity by increasing microglial necrosis and triggering microglial apoptosis (Fig. 4F). These data are in agreement with the previously reported role for RhoA in regulating microglial necrosis in steady-state conditions [24] and further demonstrate that in the absence of RhoA, neuroinflammation exacerbates microglial cell death.

**Fig. 5:**
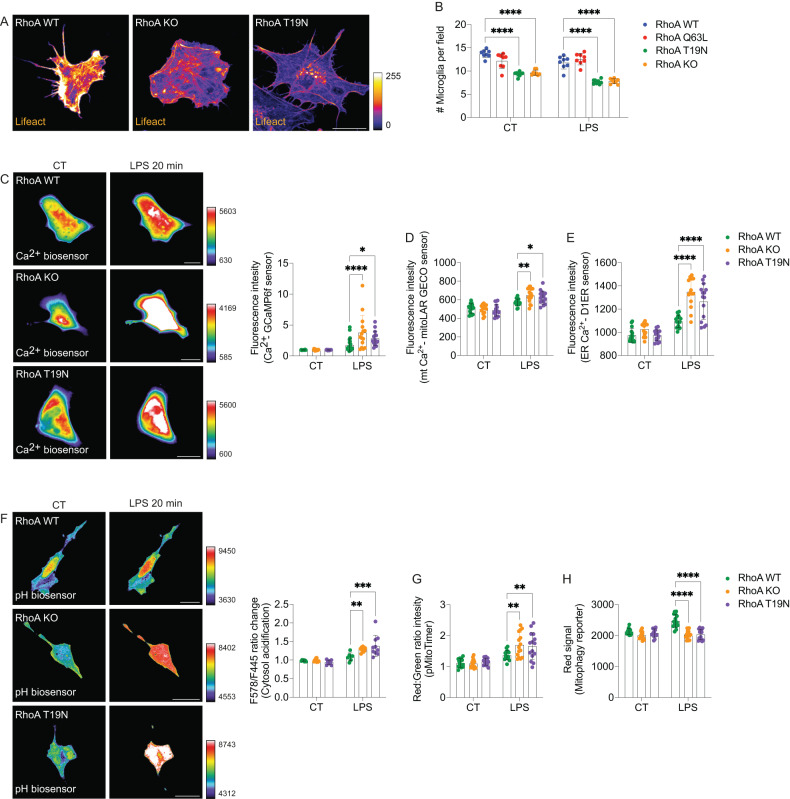
Inflammation disrupts Ca^2+^, pH, and mitochondrial homeostasis in RhoA-deficient microglia. **A** Lifeact fluorescence labeling in RhoA WT, RhoA T19N, or RhoA KO HMC3 microglia (*n* = 3 independent experiments). **B** Cell counting in RhoA WT, RhoA Q63L, RhoA T19N, or RhoA KO HMC3 microglia (*n* = 8 independent experiments). Graph displays mean with SD. ****p < 0.0001 (Two-way ANOVA). RhoA WT, RhoA T19N, or RhoA KO HMC3 microglia expressing a global Ca^2+^ biosensor (**C**), mitochondrial Ca^2+^ biosensor (**D**), endoplasmic reticulum Ca^2+^ biosensor (**E**), pH biosensor (**F**), MitoTimer biosensor (**G**), or mitophagy biosensor (**H**) and exposed to LPS (1 µg/ml; 20 min (**C-F**) or 60 min (**G and H**)). Graphs (mean and SD) display fluorescence changes (*n* = 15 cells per group from 3 independent experiments for each biosensor). *p < 0.05, **p < 0.01, ***p < 0.001, ****p < 0.0001 (Two-way ANOVA). Scale bars: 20 µm.

Afterward, we questioned which cellular mechanisms/pathways could be altered, potentially dampening microglial viability during exposure to LPS. We used two loss-of-function approaches to reduce RhoA activity in vitro substantially. In the first one, we overexpressed a dominant-negative RhoA mutant (RhoA T19N) in HMC3 microglia [24], whereas, in the second, we knocked out RhoA in HMC3 microglia (RhoA KO), using the CRISPR/Cas9 system [58]. As a functional readout for reduced RhoA activity, we measured the f-actin content in HCM3 microglia in those conditions (Fig. 5A). Confirming previous data [24, 58], microglia overexpressing RhoA T19N or RhoA KO microglia displayed decreased f-actin (lifeact labeling) content compared to microglia overexpressing RhoA WT (Fig. 5A). In agreement with the in vivo data, we observed increased cell loss in RhoA T19N or RhoA KO HCM3 microglial cultures compared to RhoA WT cultures, which was exacerbated by LPS exposure (Fig. 5B). Cell numbers were comparable between RhoA Q63L and WT HCM3 microglial cultures both in control and LPS-treated conditions (Fig. 5B).

To study the toxic effect observed in microglia lacking RhoA during inflammation, we evaluated Ca^2+^ signaling, pH equilibrium, and mitochondrial function as major parameters regulating microglial survival. Because, when dysregulated, these parameters compromise cell viability [59, 60], they might be essential hallmarks associated with microglial cell death upon RhoA ablation during neuroinflammation.

Necrotic cell death is classically associated with intracellular Ca^2+^ overload, which often cooperates with activating proteases (e.g., caspases and calpains) and endonucleases involved in apoptosis [60]. Thus, RhoA WT, T19N, and RhoA KO HCM3 microglia were co-transfected with a Ca^2+^ biosensor and exposed to LPS [61]. As expected, following LPS treatment, RhoA WT microglia exhibited a slight but consistent increase in the intracellular Ca^2+^ levels (a typical operational response) (Fig. 5C). However, both RhoA T19N and RhoA KO cells showed a further increase in intracellular Ca^2+^ following LPS stimulation compared to RhoA WT cells (Fig. 5C). Such intracellular Ca^2+^ overload likely resulted from combined dysregulation of mitochondrial and endoplasmic reticulum (ER) Ca^2+^ signaling as RhoA T19N or RhoA KO cells displayed aberrant Ca^2+^ flux in the presence of LPS in both the mitochondrial and ER compartments (Fig. 5D and E).

Intracellular pH is also tightly regulated in mammalian cells. Such stringent control relies on pH variability across different cellular organelles, which is required for their proper functions [62]. Generally, apoptotic and necrotic cell death is associated with cytoplasmic acidification [59]. This occurs mainly after caspase activation, leading to mitochondrial dysfunction and impairment of ion transporters [63, 64], culminating in abnormal H^+^ accumulation, organelle damage, and, eventually, cell death. Therefore, pH dysregulation could also be involved in the toxicity observed in RhoA-deficient microglia during LPS exposure. We monitored pH homeostasis using a genetically encoded pH sensor from mutagenesis of the red pH-sensitive protein – mKeima [43]. Like the Ca^2+^ data, we used live-cell imaging in HCM3 microglia with reduced RhoA activity (i.e., RhoA T19N and RhoA KO cells). RhoA WT microglia underwent subtle cytosolic acidification following LPS exposure (Fig. 5F). However, LPS further increased cytosolic acidosis in RhoA KO or RhoA T19N microglia (Fig. 5F).

Ca^2+^ overload and abnormal cytosolic acidification can disrupt mitochondrial function, ultimately leading to cell death. Thus, we asked whether reduced RhoA activity during inflammation would impact mitochondrial damage in microglia. To assess mitochondrial oxidation/damage, we used the fluorescent sensor pMitoTimer [65]. Following LPS treatment, RhoA WT HCM3 microglia exhibited a slight but consistent increase in the rate of oxidized-to-normal (Red:Green) mitochondria (Fig. 5G), indicating high mitochondrial activity to cope with microglial inflammatory activation. However, compared to RhoA WT, RhoA T19N or RhoA KO microglia displayed a significant increase of mitochondrial oxidation/damage upon LPS exposure (Fig. 5G), suggesting that inflammation leads to excessive/abnormal accumulation of oxidized mitochondria in the absence of RhoA.

Mitophagy eliminates oxidized/damaged mitochondria and is an essential quality control mechanism that couples cellular energetics and cell viability in stressful conditions (such as during inflammation). Thus, we measured mitophagy using a fluorescent mitophagy reporter (pCLBW cox8 EGFP mCherry [66]) in RhoA WT, T19N, and RhoA KO HCM3 microglia following inflammation. As expected, after LPS treatment, RhoA WT microglia exhibited a robust increase in mitophagy (Fig. 5H). Conversely, inhibiting RhoA activity — by overexpressing RhoA T19N or knocking out RhoA — significantly prevented the LPS-induced mitophagy (Fig. 5H).

## Discussion

Neuroinflammation is an immune response that occurs in nervous tissue and is primarily mediated by resident glial cells and circulating immune cells. Microglia are the largest population among the different types of immune cells in the brain. Microglia play a pivotal role in initiating and regulating neuroinflammation by producing cytokines, chemokines, and reactive oxygen species (ROS), which can activate or recruit other immune cells to the site of injury or infection [5]. While transient neuroinflammation is beneficial, and the mediators acutely released by microglia may contribute to tissue defense and repair [2, 3], sustained neuroinflammation is believed to cause brain damage [4].

Systemic LPS administration, which is widely used in experimental organisms (including mice) to model neuroinflammation [67], induces a neuroinflammatory response in microglia via TLR4 activation [7, 8]. Many studies, including transcriptomic data, show that LPS binding to TLR4 leads to the activation of several inflammation-related signaling cascades, including NF-κB and MAPK/AP-1 pathways, inducing the production of proinflammatory cytokines (IL-1β, IL-6, and Tnf) and interferon regulatory factors that orchestrate the neuroinflammatory response [26, 68].

Neuroinflammation is an immune response that occurs in nervous tissue and is primarily mediated by resident glial cells and circulating immune cells. Microglia are the largest population among the different types of immune cells in the brain. Microglia play a pivotal role in initiating and regulating neuroinflammation by producing cytokines, chemokines, and reactive oxygen species (ROS), which can activate or recruit other immune cells to the site of injury or infection [5]. While transient neuroinflammation is beneficial, and the mediators acutely released by microglia may contribute to tissue defense and repair [2, 3], sustained neuroinflammation is believed to cause brain damage [4].

Systemic LPS administration, which is widely used in experimental organisms (including mice) to model neuroinflammation [67], induces a neuroinflammatory response in microglia via TLR4 activation [7, 8]. Many studies, including transcriptomic data, show that LPS binding to TLR4 leads to the activation of several inflammation-related signaling cascades, including NF-κB and MAPK/AP-1 pathways, inducing the production of proinflammatory cytokines (IL-1β, IL-6, and Tnf) and interferon regulatory factors that orchestrate the neuroinflammatory response [26, 68].

Following exposure to LPS, we observed a complete shift of the RhoA activity pattern in microglia in vivo and in vitro. Such alteration indicates that the microglial response to an inflammatory stimulus is associated with the modulation of RhoA activity and suggests that RhoA might play a critical role in this process. Our observations suggest a direct relationship between TLR4 and RhoA, which is a reasonable premise given the classical association of membrane receptors with Rho GTPases and the many pathways in which RhoA appears to be involved. In agreement with this, a study in human monocytes shows that LPS modulates RhoA activity via the interleukin-1 receptor-associated kinase (IRAK) [69]. RhoA also participates in macrophage inflammatory response by regulating TLR4 signaling and internalization via p120-catenin [70]. However, the mechanism by which TLR4 controls the activity of RhoA in microglia requires further investigation.

Studies in neutrophils [71], monocytes [69], macrophages [72, 73], and BV2 microglial cell line [74, 75] report an association between TLR4 engagement and RhoA. Still, the reports suggest increases in RhoA activity in all these cases, contrasting with the decrease we observed in microglia. The modulation of RhoA activity seems cell-specific and may have distinct functional effects among immune cells. Accordingly, conditional-gene targeting studies demonstrate that many pathways a given Rho GTPase regulates are cell-type and stimulus-specific. Hence, knowing the role of RhoA in a given cell type does not necessarily predict its function and signaling mechanisms in another [76].

As inflammation decreased microglial RhoA activity, we asked whether sustained RhoA activity would prevent LPS-mediated inflammation. Thus, we overexpressed a constitutively active RhoA mutant (RhoA Q63L) in microglia. Overexpressing RhoA Q63L suppressed two critical hallmarks of microglial inflammation: metabolic reprograming and proinflammatory polarization.

In immune cells, including microglia, inflammatory polarization requires a metabolic reprogramming [14, 15] characterized by a shift from oxidative phosphorylation (OXPHOS) to aerobic glycolysis [29, 77, 78]. Although glycolysis is less efficient than OXPHOS when it comes to ATP production, the rate of glucose metabolism is much faster, fueling energy-intensive processes and enabling an immediate cellular response [79]. In agreement with our data, the change to a glycolytic metabolism implies a decrease in the ATP:ADP ratio, which results from the negative balance between low ATP production and high glucose consumption [80], which leads to the accumulation of pyruvate and lactate. In line with the literature [81, 82], we showed that using FRET-based nanosensors and Seahorse-based bioenergetic profiling, microglia exposed to LPS became glycolytic. However, sustained microglial RhoA activity prevented this metabolic shift, indicating that a decrease in RhoA activity during inflammation is critical for the metabolic reprogramming required for microglia proinflammatory polarization.

Accordingly, we found that sustained RhoA activity during LPS stimulation arrested proinflammatory polarization, associated with inhibition of ERK and Src activation (typical protein kinases involved in LPS-mediated microglia proinflammatory polarization [37, 83]). ERK and Src converge to activate the NF-κB pathway, producing proinflammatory cytokines (including TNF and IL-1ß). Thus, the attenuation of ERK and Src activation likely compromised the production of those factors. The production of ROS and the decrease of AMPK activation are two other classical features of LPS-mediated microglia inflammation that were significantly inhibited by sustained RhoA activity. The RhoA/ROCK pathway is associated with NADPH oxidase, a major ROS generator in the microglia [74, 84]. Regarding AMPK, its activation is sufficient to reduce LPS-induced inflammation in microglia [42], and various compounds that activate AMPK were tested in LPS-stimulated microglia to regulate neuroinflammation [85, 86]. Thus, our data suggest that decreasing RhoA activity modulates different pathways associated with microglia’s proinflammatory capacity.

To further characterize the bona fide role of RhoA in microglia during neuroinflammation, we used a microglia-specific conditional Cre line based on the insertion of the Cre^ERT2^ cassette into the Cx3cr1 locus [57]. As before, we conditionally ablated RhoA in microglial cells [24] by crossing Cx3cr1^CreER-eYFP^ [57] mice with RhoA floxed mice [87].

We previously reported that loss of RhoA in steady-state conditions is sufficient to increase microglial necrosis but not apoptosis [24]. Here, we found that a single LPS administration in mice with RhoA-deficient microglia induced an acute neuroinflammatory condition, worsening microglial necrosis and eliciting microglial apoptosis to a lesser extent. This cytotoxic effect of neuroinflammation in microglia lacking RhoA was corroborated using analyses of microglial morphology in which we found clear hallmarks of cell damage, including process loss/beading and dysmorphic cell shape accompanied by cell body swelling [88–90]. Considering that RhoA ablation causes microglial necrosis in steady-state conditions, we concluded that in the absence of RhoA, neuroinflammation aggravates microglia cell death by exacerbating necrosis and triggering apoptosis.

Our data shed some light on the mechanisms underlying the toxicity of the lack of RhoA in microglia during inflammation. Upon LPS exposure, there was a disruption of Ca^2+^, intracellular pH, and mitochondrial homeostasis in RhoA-deficient microglia. The molecular links between RhoA activity and Ca^2+^ signaling are yet to be identified in microglia. In Hela, MDKC, RP1, and HEK293T cells, optogenetic activation of RhoA at the cell edge in the steady-state is sufficient to increase Ca^2+^ transients [91], which is the opposite of what we showed herein during inflammation, suggesting that RhoA-to-Ca^2+^ signaling is likely cell-type and context-specific. Moreover, changes in the status of microglia reactivity elicited by the loss of RhoA are mimicked by the ablation of type II myosin [58], a major RhoA downstream effector involved in regulating Ca^2+^ homeostasis in different cell types [91]. Although more studies are needed to pinpoint the specific molecular links between RhoA and Ca^2+^ in microglia, it is possible that upon loss of RhoA, myosin II-dependent inflammation dysregulates ion transporters (e.g., ATPases or Na^2+^/Ca^2+^ exchangers), leading to cytosolic Ca^2+^ and H^+^ overload. Excessive Ca^2+^ and H^+^ may lead to the activation of proteases, endonucleases, and phospholipases involved in the cleavage of organellar membranes, culminating in mitochondrial damage and dysfunction [60].

An increased number of damaged mitochondria is a significant hallmark of many forms of cell death, including necrosis and apoptosis. In microglia expressing RhoA, inflammation increased mitochondrial oxidation/damage paralleled by mitophagy (an essential quality control mechanism for removing damaged mitochondria). In microglia lacking RhoA, inflammation increased mitochondrial damage even further without enhancing mitophagic flux. This suggests that the higher accumulation of damaged mitochondria in RhoA-deficient microglia likely resulted from mitophagy impairment. Thus, mitochondrial dysfunction (triggered by disruption of Ca^2+^ and pH homeostasis) and insufficient mitophagy may accelerate microglial cell death during neuroinflammation.

**Fig. 6:**
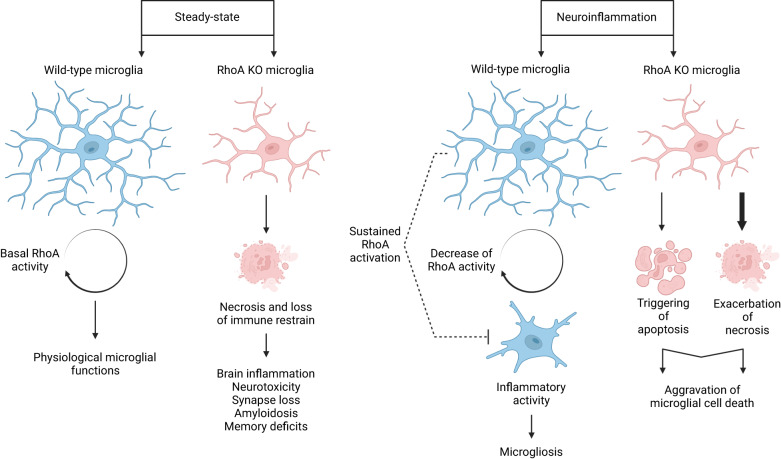
Microglial reaction to neuroinflammation requires tight control of RhoA activity. LPS-induced microglia inflammatory response requires a decrease in RhoA activity so that sustaining it prevents overall microglia inflammatory reaction. On the other hand, in RhoA KO microglia, LPS-induced inflammation causes apoptosis and worsens necrosis, likely through dysregulation of Ca^2+^, pH, and mitochondrial homeostasis.

Under steady-state conditions, the loss of RhoA-dependent immune regulation results in microglial necrosis and neurotoxicity, which can cause brain inflammation, synapse loss, amyloidosis, and memory deficits (Fig. 6 — vide [24]). During inflammation, RhoA deficiency can worsen microglial necrosis and trigger apoptosis, leading to increased microglial cell death (Fig. 6). We suggest that decreasing RhoA activity (to a certain extent) is necessary to achieve adequate microglial inflammatory capacity. However, maintaining a minimum level of active RhoA is essential for the survival of microglia during inflammation. Therefore, fine-tuning RhoA signaling to balance microglial reactivity may be beneficial for preserving neuronal plasticity in brain diseases that involve neuroinflammation.

## Materials and methods

### Animals

Experiments using mice were approved by Direção Geral de Alimentação e Veterinária (DGAV) and by the animal ethics committee of IBMC-i3S, Porto. Animal facilities and the people directly involved in animal experimentation were also certified by DGAV. All animal experiments considered the Russell and Burch 3 R’s principle and followed the European guidelines for animal welfare (2010/63/EU Directive), ensuring minimal animal suffering. Animals were maintained in standard laboratory conditions with a 12 h/12 h light/dark cycle and were allowed free access to food and water.

Wild-type mice were bred and maintained at the i3S animal facility. Conditional microglia RhoA-deficient mice were generated as before [24] using two different mice strains: Cx3cr1^CreER-EYFP^ mice (purchased from Jackson Laboratories; stock number: 021160), in which the Cx3cr1 promoter drives high expression of the CreER cassette in microglia [57] and mice homozygous for the RhoA floxed allele. The progeny of interest were RhoA^fl/fl^ (controls) and RhoA^fl/fl^:Cx3cr1^CreER+^ (conditional KOs). To conditionally ablate RhoA in microglia, RhoA^fl/fl^ and RhoA^fl/fl^:Cx3cr1^CreER+^ mice were given tamoxifen as before [24, 92]. We kept mice on a C57Bl/6 background in all experiments. This study included both males and females.

Genotype determination was performed by PCR on genomic DNA. Primers used for RhoA floxed alleles were AGC CAG CCT CTT GAC CGA TTT A (forward); TGT GGG ATA CCG TTT GAG CAT (reverse). Primers for CreERT2 insertion were: AAG ACT CAC GTG GAC CTG CT (WT forward); AGG ATG TTG ACT TCC GAG TG (WT reverse); CGG TTA TTC AAC TTG CAC CA (mutant reverse).

### LPS administration

RhoA^fl/fl^ and RhoA^fl/fl^:Cx3cr1^CreER+^ mice (100-110 day-old) were intraperitoneally injected with LPS (4 mg/kg) from Escherichia coli 0111:B4 (Sigma Aldrich). Both genotypes were also similarly administered with a saline solution (NaCl). Twenty-four hours post-administration, animals were sacrificed and had their brains harvested for different analyses.

### Flow cytometry and cell sorting

To identify microglia, we used CD45-PE (103106 BioLegend) and CD11b-APC (101212 BioLegend, USA) as we did before [24, 92]. Necrotic and apoptotic microglia were determined using Annexin V (640906 BioLegend, USA) and Zombie Violet Dye (77477 BioLegend, USA).

Mice were anesthetized with sodium pentobarbital (0.2 ml *per* 30 g of mice body weight) and then perfused (transcardial perfusion) with ice-cold PBS. Brains were harvested, placed on ice-cold Gibco^®^ RPMI 1640 (Thermo Fisher, USA), and mechanically homogenized to obtain single-cell suspensions. The cell suspension was passed through a 100μm cell strainer and centrifuged over a discontinuous 70/30% Percoll (Sigma-Aldrich, USA) gradient for 30 min. Cells on the interface were collected, pelleted, resuspended in FACS buffer (2% BSA; 0.1% Sodium Azide in PBS), and then counted on Countess^TM^ automated cell counter (Thermo Fisher) using trypan blue exclusion to estimate the number of live cells. A single cell suspension (5 × 10^5^ cells) was incubated with the different FACS antibodies for 30 min at 4 °C in the dark. Compensation settings were determined using spleen from both control and RhoA cKO mice. A FACS Canto II analyzer (BD Immunocytometry Systems, USA) was used to evaluate cell suspensions. All data were analyzed by FlowJo X10 software^®^ (TreeStar, USA) using a sequential gating strategy. Sorting of microglia from RhoA^fl/fl^ and RhoA^fl/fl^:Cx3cr1^CreER+^ brains was performed as before [24]. Sorting of HCM3 microglia expressing GFP-RhoA WT and GFP-RhoA T19N was performed as before [58].

### Gene expression

RNA was extracted from microglia using the Direct-zol RNA Miniprep Kit. Complementary DNA synthesis was performed using 500 ng of total RNA [deoxyribonuclease I (DNase I)–treated] with SuperScript III First-Strand Synthesis SuperMix. Quantitative reverse transcription (qRT)–PCR was carried out using iQ SYBR Green Supermix on an iQ5 multicolor real-time PCR detection system (Bio-Rad). The expression of PCR transcripts was calculated using the 2 − Δ*C*t with *Yhwaz* serving as the internal control gene. Statistical analyses were performed on raw 2 − Δ*C*t values using unpaired *t-tests* to detect differentially expressed transcripts between sampled groups.

### Brain tissue preparation and immunolabeling

After animal perfusion with ice-cold PBS (15 ml), brains were fixed by immersion in 4% PFA in PBS, pH 7.2 overnight. After that, brains were washed with PBS and then cryoprotected using gradually increased sucrose concentrations in a row (15 and 30% w/v). After 24 h, brains were embedded in an OCT medium, frozen (−80 °C), and cryo-sectioned in the CM3050S Cryostat (Leica Biosystems, Germany). Coronal sections from brains (30μm thickness) were collected non-sequentially on Superfrost Ultra Plus^®^ slides. Brain sections from RhoA^fl/fl^ and RhoA^fl/fl^:Cx3cr1^CreER+^ mice (administered with LPS or NaCl) encompassing identical stereological regions were placed side by side on the same glass slide. Slides were stored at −20 °C until processed for immunolabeling. Frozen sections were thawed for at least 1 h and hydrated with PBS for 15 min. Sections were permeabilized with 0.25% Triton X-100 for 15 min, washed with PBS for 10 min, and blocked (5% BSA, 5% FBS, 0.1% Triton X-100) for 1 h. Primary antibody anti-Iba1 (1:500; RRID:AB_839504) and anti-Active-RhoA GTP (1:100; RRID:AB_1961799) were incubated in blocking solution in a humidified chamber overnight at 4 °C. The secondary antibodies AlexaFluor 594 or AlexaFluor 647 (1:500) were incubated for 2 h in a blocking solution. After the secondary antibody, sections were washed three times for 10 min with PBS and rinsed twice in PBS. Slides were coverslipped using Fluoroshield^TM^ (Sigma Aldrich) and visualized under a Leica TCS SP5 II confocal microscope.

### Confocal imaging reconstruction and analyses

Images from tissue sections (cortex region) were acquired using a Leica HC PL APO Lbl. Blue 20x /0.70 IMM/CORR water objective in 8-bit sequential mode using standard TCS mode at 400 Hz, and the pinhole was kept at one airy in the Leica TCS SP5 II confocal microscope. Images were resolved at 1024 × 1024 pixels format illuminated with 2–5% DPSS561 561 nm wave laser using a HyD detector in the BrightR mode, and the entire Z-series were acquired from mouse brain sections. Images with equivalent stereological regions were obtained for all tissue sections within a given slide. Image series were deconvolved using the Hyugens Professional using the Classic Maximum Likelihood Estimation (CMLE) algorithm. A determined theoretical PSF was established using a routine-based implementation for the Hyugens software. Images from different sections within a given slide were acquired on the same day, always by the same operator, and with identical microscope parameters (e.g., same laser-line potency; same power for the confocal laser lines; same objective; exact fluorescence exposure times and offset for a given fluorophore; same pinhole aperture; same, zoom and ROI magnification; same pixel size; same TCS scanner mode and speed; same z-stack step size and optical sectioning and same line averaging).

To quantify GTP-RhoA in microglia, images from stereologically identical brain regions (4 images per section; 3 sections per animal for each experimental group) were acquired from each experimental group. Using FIJI software, confocal *Z* stacks were background-subtracted and smoothened using a Sigma-Aldrich filter. GTP-RhoA and Iba1 volumes were reconstructed using 3D surface rendering of confocal *Z* stacks in Imaris as before [92, 93].

### Primary cultures of cortical microglia

Primary microglial cell cultures were performed as previously described [51, 94]. In brief, mice pups (2-day-old) were sacrificed, and their cerebral cortices were dissected in HBSS, pH 7.2, and digested with 0.07% trypsin plus 50 μL (w/v) DNAse for 15 min. Next, cells were gently dissociated using a glass pipette in DMEM F12 GlutaMAX™-I (Thermo Fisher) supplemented with 10% FBS, 0.1% gentamicin. Cells were plated in polyD-lysine-coated T-flasks (75 cm^2^) at 1.5 × 10^6^ cells per cm^2^. Cultures were kept at 37 °C and 95% air/5% CO_2_ in a humidified incubator. Culture media was changed every 3 to 4 days up to 20 days. Culture flasks were subjected to orbital shaking at 200 rpm for 2 h to obtain purified microglial cell cultures. Next, the culture supernatant was collected and centrifuged at 453 g for 5 min at room temperature. The supernatant was discarded, and the microglia pellet was resuspended in a culture medium. Ultimately, cells were seeded in poly-D-lysine-coated six or 12-well culture plates at 2.5 × 10^5^ cells/cm^2^ with Dulbecco’s Modified Eagle Medium (DMEM) F12 + GlutaMAX™-I (Thermo Fisher) supplemented with 10% FBS, 0.1% gentamicin and one ng/ml M-CSF or one ng/ml GM-CSF. Purified microglia were cultured for 5–8 days. Primary microglia were transfected as before [51, 94].

For dihydroethidium measurements, cells were transfected with different RhoA constructs and allowed to recover for 3 days. Then, cultures were loaded with dihydroethidium 5 µM for 30 min before incubation with control saline or LPS (1 µg/ml) for 24 h. Cultures were fixed with PFA 4%, and fluorescence was measured in a plate reader.

### Microglial cell line

The human microglia clone 3 (HMC3) cell line was obtained through SV40-dependent immortalization of human embryonic microglial cells and authenticated by the American Type Culture Collection (ATCC^®^ CRL-3304^™^). These cells were cultivated with DMEM + GlutaMAX^™^-I (supplemented with 10% FBS and 1% Penicillin/Streptomycin) and maintained at 37 °C, 95% air, and 5% CO2 in a humidified incubator. RhoA KO HMC3 microglia cell line, obtained using CRISPR-Cas9 technology, was generated as before [58]. Cell counting was performed after the incubation of cultures with LPS for 24 h. Culture dishes were washed in excess PBS and fixed with 4% PFA in phosphate buffer 0.2 M for 10 min. Cell numbers in each condition were determined by counting ten to twelve randomly selected microscopic fields using a Leica DMI6000B inverted microscope.

### Fluorescence imaging in cultured microglia and quantifications

For quantifications in primary and HCM3 microglia, images were exported as raw 16-bit tiff using the LAS AF software. The background was subtracted in FIJI using the roller-ball ramp between 35–50% pixel radius. Images were segmented in FIJI using the local Otsu threshold. Thresholded images were converted to binary masks using the dark background function. Binary mask images were multiplied for their original channel images using the image calculator plugin to generate masked 32-bit float images relative to each channel. Original coordinate vectors were retrieved from the ROI manager, and FIJI returned the mean fluorescent intensity in gray values contained within any single microglia using the multi-measure function. The mean fluorescent intensity for every microglia was exported and statistically evaluated using GraphPad Prism^®^ software.

### Live-cell imaging and analyses of biosensors

Primary or HMC3 microglia were plated at a density of 25000 cells/dish on plastic-bottom culture dishes (µ-Dish 35 mm, iBidi) with Dulbecco’s Modified Eagle Medium (DMEM) + Glutamax^®^ (supplemented with 5% FBS and 1% Penicillin/Streptomycin). Cells were transfected with the different biosensors using the JetPrime DNA transfection reagent (Polyplus Transfection SA., USA) in a proportion of 2 µL of reagent per 1 µg of DNA. The total medium was changed 4 h after transfection.

Imaging was performed 24 h post-transfection using a Leica DMI6000B inverted microscope. During the assay, cells were kept under 37 °C in HBSS with CaCl_2_ and MgCl_2_ (Thermo Fisher) buffered with HEPES 15 mM (Thermo Fisher) except for cells transfected with Ca^2+^ and pH biosensors where the imaging was performed using DMEM D-Glucose, L-Glutamine, HEPES without phenol red (Thermo Fisher). Cells were recorded before (5 min; baseline) and after that stimulation with LPS.

The excitation light source was a mercury metal halide bulb integrated with an EL6000 light attenuator. Images were acquired using a PlanApo 63 × 1.3NA glycerol immersion objective. High-speed, low-vibration external filter wheels (equipped with CFP/YFP excitation and emission filters) were mounted on the microscope (Fast Filter Wheels, Leica Microsystems). CFP-YFP FRET filter set consisted of CFP (Ex: BP 427/10; Em: BP 472/30), YFP (Ex: BP 504/12; Em: BP 542/27), and a 440–520 nm dichroic mirror (CG1, Leica Microsystems). An RFP filter cube (TX2 Excitation: 560/40; BS: 595; Emission: 645/75) was used for red fluorescence images. Images were acquired with 2 × 2 binning using a digital CMOS camera (ORCA-Flash4.0 V2, Hamamatsu Photonics). At each time point, images were sequentially acquired using different filter combinations according to each biosensor. Quantification of biosensors was performed using FIJI software. Briefly, images were exported as 16-bit tiff files to the software, and the background was dynamically removed from all frames from both channels. Ratiometric images were generated using the PFRET plugin for ImageJ. Finally, whole-cell analyses were performed, and the mean values for each time point were extracted. Supplementary Figure 2 includes positive controls for the FRET biosensors for detecting RhoA, Src, and ERK.

### Seahorse assay

Seahorse assay was performed using an XF24 extracellular flux analyzer (Agilent Technologies) to determine the bioenergetic profile of intact HCM3 microglia. GFP-RhoA WT or GFP-RhoA Q63L HCM3 cells were flow cytometry-sorted, harvested, and seeded onto poly-d-lysine-coated XF24 plates at a density of 10^4^ cells per well. The cells were then incubated in DMEM medium supplemented with 5 mM glucose, 2 mM glutamine, and 10% FBS for 48 h at 37 °C and 5% CO^2^. The Seahorse Mitochondrial Respiration Kit was used according to the manufacturer’s instructions to assess mitochondrial respiration and energy metabolism. The oxygen consumption rate (OCR) was measured under basal conditions and after sequential treatments with vehicle or 1 µg/ml lipopolysaccharide (LPS) for cell activation; 1.5 μM oligomycin for ATP synthase inhibition; 1 μM carbonyl cyanide-4-(trifluoromethoxy)phenylhydrazone (FCCP) for mitochondrial uncoupling; 0.5 μM rotenone/antimycin A for mitochondrial complex I and III inhibition. The XF24 extracellular flux analyzer recorded the OCR measurements at each step. The bioenergetic profile of the HCM3 microglia cells was calculated using cloud-based Agilent Seahorse Analytics (Agilent).

### Cell lysates and Western blotting

Cell cultures were lysed using RIPA-DTT buffer (150 mM NaCl, 50 mM Tris, 5 mM EGTA, 1% Triton X-100, 0.5% DOC, 0.1% SDS) supplemented with complete-mini protease inhibitor mix, 1 mM DTT and phosphatase inhibitor cocktail. Samples were sonicated (7 pulses of 1 sec at 60 Hz) and centrifuged at 16,000 g, 4 °C for 10 min. After the supernatant collection, protein concentration was determined by the BCA method. All samples were denatured with sample buffer (0.5 M Tris-HCl pH 6.8, 30% glycerol, 10% SDS, 0.6 M DTT, 0.02% bromophenol blue) at 95 °C for 5 min and stored at −20 °C until use.

Samples were separated by SDS-PAGE gel electrophoresis with a voltage of 120 V (adjustments over time were made). Precision Plus Protein™ Dual Color Standards (1610374; Bio-Rad) was used as a molecular weight marker. Proteins were transferred from gel to the Immun-Blot^®^ PVDF membrane (Bio-Rad) using a Trans-Blot^®^ Turbo™ Transfer System (Bio-Rad). The transference was performed for 10 min in diluted Trans-Blot^®^ Turbo™ 5X Transfer Buffer (20% buffer, 20% ethanol, and 60% ultrapure H_2_O). Membranes were blocked for 60 min in a blocking solution composed of 5% skimmed milk diluted in tris-based saline with 0.1% Tween (TBS-T) pH 7.6 and incubated with primary antibodies diluted in a blocking solution overnight at 4 °C. Membranes were washed three times, for 10 min each, with TBS-T and incubated with peroxidase-conjugated secondary antibodies: HRC conjugated anti-rabbit (1:10000; Promega) or HRC conjugated anti-mouse (1:15000; Promega). Membranes were developed using a Pierce™ ECL Fast Western Kit (Thermo Fisher) and revealed using ChemiDoc™ XRS System (Bio-Rad). Images were quantified in FIJI Software^®^. Full gels are provided in the supplementary figures.

### RhoA pull-down

RhoA activity was measured using a GST-rhotekin-based assay, as described previously [22, 95]. Briefly, expression of recombinant protein GS-rhotekin was induced in transformed BL21 *Escherichia coli* by adding 0.1 M isopropylthiogalactoside for 4 h. Bacteria were harvested, resuspended in lysis buffer (50 mM Tris-HCl, pH 8.2, MgCl_2_, 0.2 mM Na_2_S_2_O, 10% glycerol, 20% sucrose, 2 mM dithiothreitol, 1 μg/ml leupeptin, 1 μg/ml pepstatin and 1 μg/ml aprotinin) and sonicated at 4 °C. Cell lysates were centrifuged for 20 min at 4 °C (45,000 g), and the cleared supernatant was stored at −80 °C. The supernatant (300 µl) was incubated with 40 µl of Glutathione High Capacity magnetic agarose beads (Sigma-Aldrich) for 30 min, at 4 °C, with gentle agitation. Beads were washed twice using lysis buffer and twice with FISH buffer (10% glycerol, 50 mM Tris-HCl, pH 7.4, 100 mM NaCl, 1% NP-40, 2 mM MgCl_2_).

Microglial cultures were homogenized in cold FISH buffer (with protease inhibitor cocktail) and centrifuged for 15 min at 4 °C (13,000 g). Precisely 10% of the volume was taken and frozen (input). The remaining supernatant was incubated with the bacterially produced GST-rhotekin fusion bound to GST-coupled magnetic beads for about 12 h, with gentle agitation at 4 °C, and washed four times with excess of FISH buffer with protease inhibitor cocktail. For elution, GLB was added, and samples were incubated at 95 °C for 10 min. Samples were resolved on a 12% SDS PAGE gel, followed by standard Western blotting for RhoA.

### Antibodies

GTP-RhoA (NewEast Biosciences Cat# 26904, RRID:AB_1961799), RhoA (Abcam Cat# ab68826, RRID:AB_1142593), RhoA (Cell Signaling Technology Cat# 2117, RRID:AB_10693922), Iba-1 (FUJIFILM Wako Shibayagi Cat# 27030, RRID:AB_2314667), phosphorylated Src family (Cell Signaling Technology Cat# 6943, RRID:AB_10013641), Src (Cell Signaling Technology Cat# 2102, RRID:AB_331358), phosphorylated ERK 1/2 (Cell Signaling Technology Cat# 9101, RRID:AB_331646), ERK 1/2 (Cell Signaling Technology Cat# 9102, RRID:AB_330744).

### Plasmids

Raichu-RhoA (provided by M. Matsuda [27]), pTriEx-RhoA FLARE.sc Biosensor WT (RRID:Addgene_12150), pTriEx-RhoA FLARE.sc Biosensor Q63L (RRID:Addgene_12151), pTriEx-RhoA FLARE.sc Biosensor T19N (RRID:Addgene_12152), pRK5-myc-RhoA WT (RRID:Addgene_12962), pRK5-myc-RhoA Q63L (RRID:Addgene_12964), pRK5-myc-RhoA T19N (RRID:Addgene_12963), EGFP-p65 (RRID:Addgene_111190), GW1-pHRed (RRID:Addgene_31473), GW1CMV-Perceval (RRID:Addgene_21737), Laconic/pcDNA3.1 (+) (RRID:Addgene_118627), Pyronic /pcDNA3.1 (+) (RRID:Addgene_51308), pcDNA3.1 FLII12Pglu-700uDelta6 (RRID:Addgene_17866), Cyto-ABKAR (RRID:Addgene_61510), pLentiEKAR2G2 (RRID:Addgene_40178), Kras-Src FRET biosensor (RRID:Addgene_78302), pFRET-HSP33 cys (RRID:Addgene_16076), pGP-CMV-GCaMP6F (RRID:Addgene_40755), mCherry-Lifeact-7 (RRID:Addgene_54491), pMitoTimer (RRID:Addgene_52659), pCLBW cox8 EGFP mCherry (RRID:Addgene_78520).

### Cytokine release

Cytokines in cell culture supernatants were quantified by enzyme-linked immunosorbent assay following the instructions provided by the manufacturer (PeproTech, UK). The culture medium was collected into tubes and centrifuged at 16,000 *g* at four °C for 5 min. The supernatant was transferred to a new tube and kept at −80 °C. A multimode microplate reader measured the absorbance at 405 nm and wavelength correction at 650 nm (Synergy HT, BioTek, USA).

### Statistical analyses

A 95% confidence interval was used, and p < 0.05 was defined as a statistically significant difference in all groups. The Mann-Whitney test, or unpaired t-test, was used to compare two experimental groups assuming equal variance among groups. Two-way ANOVA with Sidak’s multiple comparisons test was used to compare four experimental groups.

To determine the effect size for this study, we conducted a comprehensive review of previous research on microglia conducted by our lab and others. Additionally, we considered potential sources of variability that may affect the effect size and sample size estimation, such as variations in microglial activation across brain regions, gender of the mice, and experimental conditions.

In this study, mice were randomly assigned to experimental groups using simple random sampling. As such, mice were given a unique identification number, and a computer routine was used to randomly assign them to the appropriate experimental group according to the study design.

To minimize observer bias, the investigators who analyzed the data were not informed of the mice genotypes or experimental conditions.

All quantifications were performed using GraphPad Prism 9.0 (GraphPad^®^ software).

### Illustrations

The figures displayed in this work were assembled with BioRender.com and Adobe Illustrator.

### Supplementary information

Suppl fig 1

Suppl fig 2

Suppl fig 3

Legeds for the Supplementary Figures 1-3

## Data Availability

All datasets on which the conclusions rely are presented in the manuscript.
