# Correction: RhoA balances microglial reactivity and survival during neuroinflammation

**DOI:** 10.1038/s41419-023-06340-8

**Published:** 2023-12-08

**Authors:** Renato Socodato, Artur Rodrigues-Santos, Joana Tedim-Moreira, Tiago O. Almeida, Teresa Canedo, Camila C. Portugal, João B. Relvas

**Affiliations:** 1 Institute of Research and Innovation in Health (i3S) and Institute for Molecular and Cell Biology (IBMC), University of Porto, Porto, Portugal; 2 Faculty of Medicine of the University of Porto (FMUP), Porto, Portugal; 3 ICBAS - School of Medicine and Biomedical Sciences, Porto, Portugal

**Keywords:** Microglia, Cell death in the nervous system

Correction to: *Cell Death & Disease* 10.1038/s41419-023-06217-w, published online 20 October 2023

In this article, the funding note has been corrected:

This work was funded by Portuguese funds through FCT—Fundação para a Ciencia e a Tecnologia under the projects PTDC/MED-NEU/1677/2021 and EXPL/MED-NEU/0588/2021.

The original article has been corrected.

