# Supplementary figures and images for: RhoA balances microglial reactivity and survival during neuroinflammation
Source: Cell Death Dis. 2023 Oct 20;14(10):690. doi: 10.1038/s41419-023-06217-w (PMC10589285; doi:10.1038/s41419-023-06217-w)

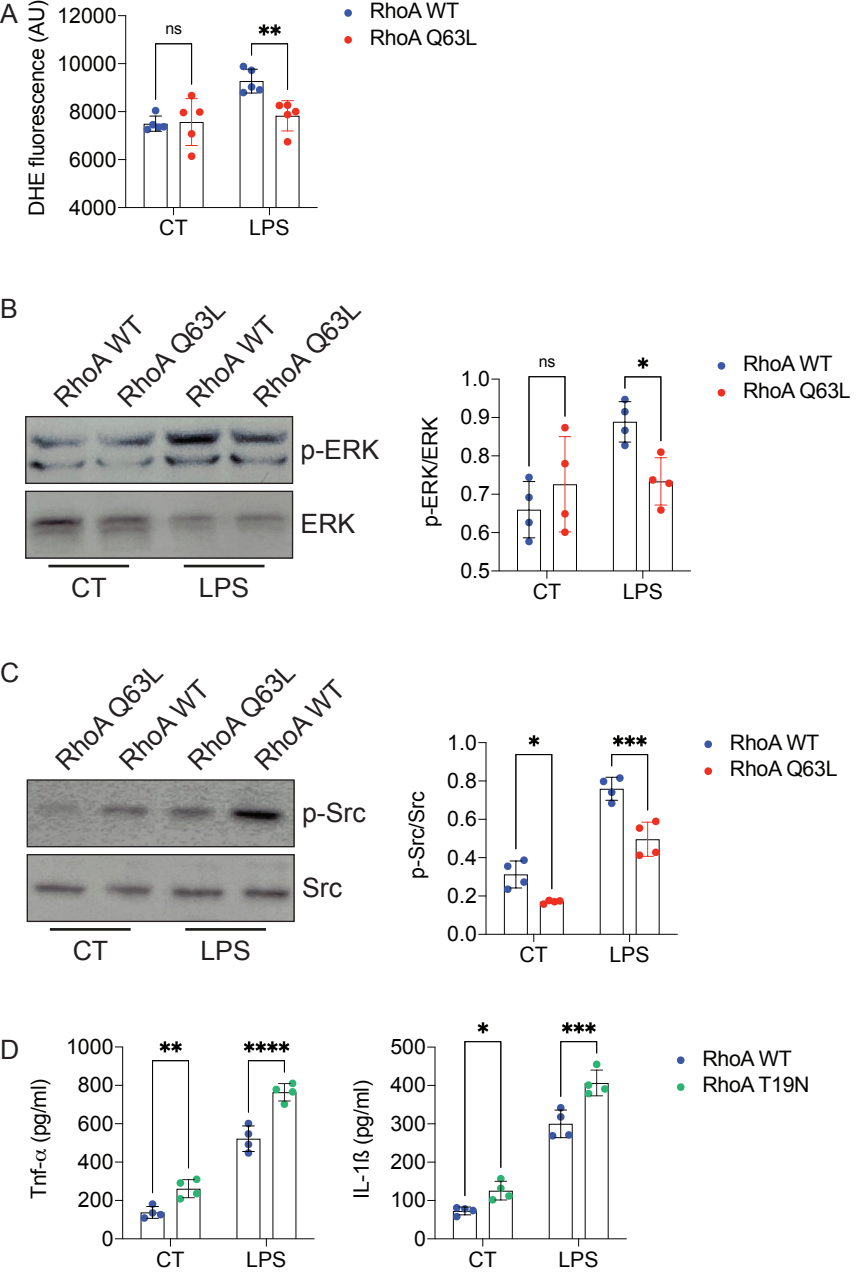

Supplement: Supplementary file 1 — Suppl fig 1 [file 41419_2023_6217_MOESM1_ESM.pdf]

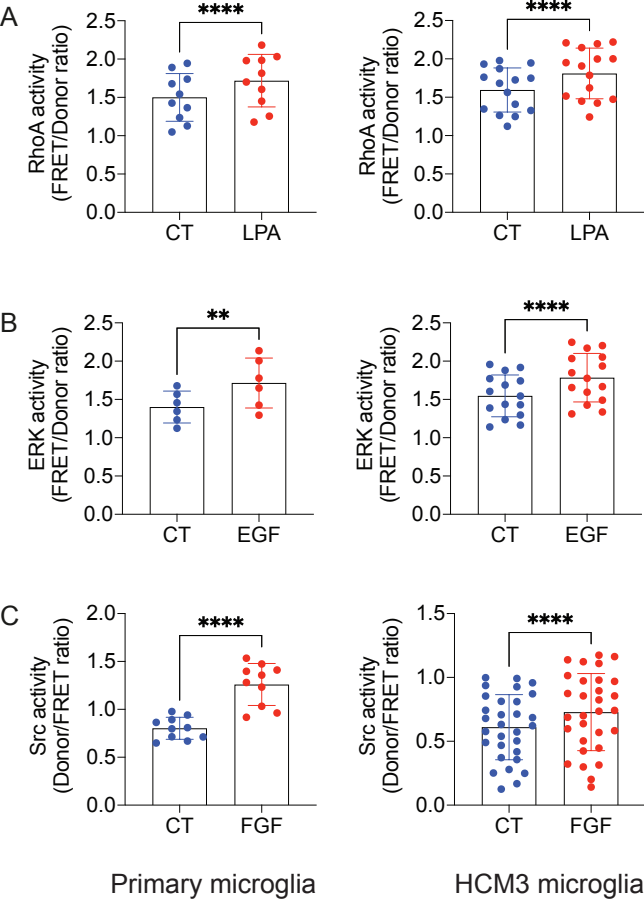

Supplement: Supplementary file 2 — Suppl fig 2 [file 41419_2023_6217_MOESM2_ESM.pdf]
