# Supplementary material for: RhoA balances microglial reactivity and survival during neuroinflammation
Source: Cell Death Dis. 2023 Oct 20;14(10):690. doi: 10.1038/s41419-023-06217-w (PMC10589285; doi:10.1038/s41419-023-06217-w)

Gel shown in Figure 1D

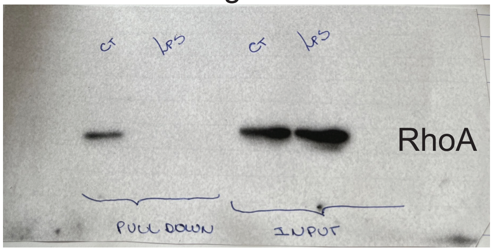

Gel shown in Figure 1F

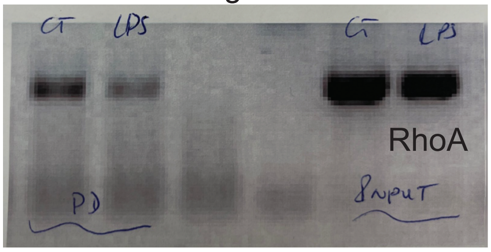

Gels shown in Suppl. Figure 1B

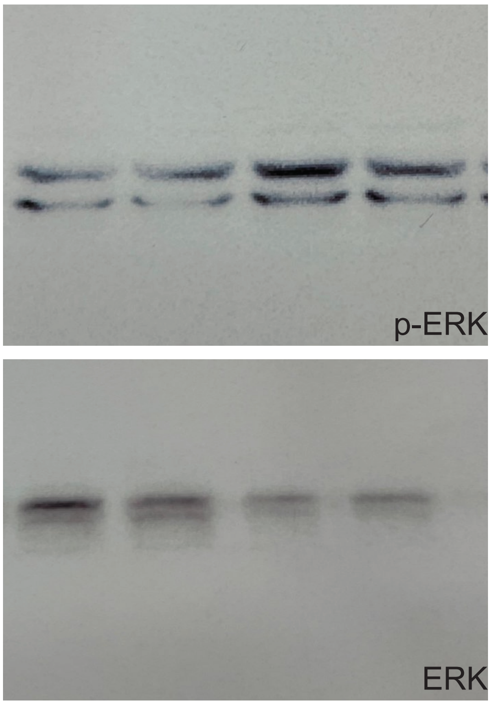

Gels shown in Suppl. Figure 1C

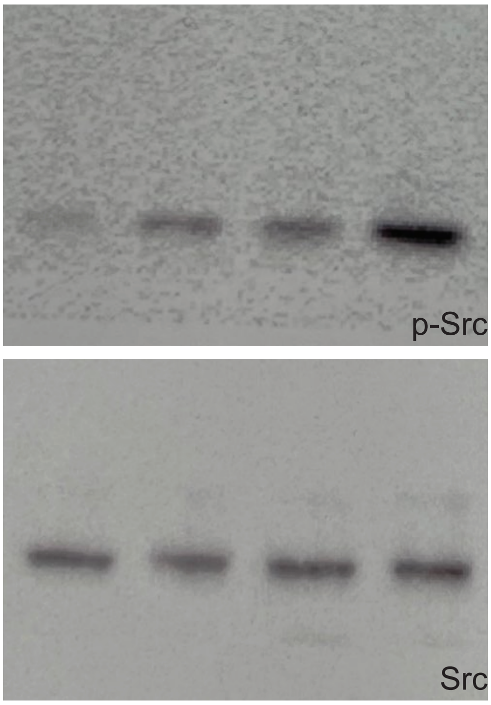

Supplement: Supplementary file 3 — Suppl fig 3 [file 41419_2023_6217_MOESM3_ESM.pdf]
