# Supplementary material for: RhoA balances microglial reactivity and survival during neuroinflammation
Source: Cell Death Dis. 2023 Oct 20;14(10):690. doi: 10.1038/s41419-023-06217-w (PMC10589285; doi:10.1038/s41419-023-06217-w)
Supplement: Supplementary file 4 — Legeds for the Supplementary Figures 1-3 [file 41419_2023_6217_MOESM4_ESM.docx]

**Supplementary Figure 1. DHE, ERK, Src, and ELISA measurements in microglia.**

(**A**) DHE fluorescence in primary cortical microglia transfected with RhoA Q63L or RhoA WT and exposed to LPS (1 µg/ml) for 24 hours (n=5 independent experiments).

(**B and C**) Western blot for ERK and Src on HCM3 microglial lysates (n=4 independent experiments).

(**D**) ELISA (TNF-α or IL-1ß) from culture supernatants of primary cortical microglia transfected with RhoA T19N or RhoA WT and exposed to LPS (1 µg/ml) for 3 hours (n=4 independent experiments).

All graphs represent means with SD. Statistical significance was determined by two-way ANOVA with *p<0.05, **p<0.01, ***p<0.001, and ****p<0.0001.

**Supplementary Figure 2. FRET positive controls in microglia.**

**A**, Primary cortical or HCM3 microglia expressing the Raichu-RhoA biosensor exposed to 10 µM lysophosphatidic acid — LPA (data was pooled across 5 independent cultures).

**B**, Primary cortical or HCM3 microglia expressing the ERK biosensor exposed to 50 ng/ml epidermal growth factor — EGF (data was pooled across 3 independent cultures).

**C**, Primary cortical or HCM3 microglia expressing the Src biosensor exposed to 50 ng/ml fibroblast growth factor — FGF (data was pooled across 3 independent cultures).

Exposure times were 20 minutes for LPA, EGF, and FGF. Graphs (mean and SD) display FRET/CFP or CFP/FRET ratio changes. Statistical significance was determined by paired t-test with **p<0.01 and ****p<0.0001.

**Supplementary Figure 3. Uncropped Western blot gels.**
